# Compositional and Functional Analysis of the Microbiome in Tissue and Saliva of Oral Squamous Cell Carcinoma

**DOI:** 10.3389/fmicb.2019.01439

**Published:** 2019-06-26

**Authors:** Zhen Zhang, Junjie Yang, Qiang Feng, Bin Chen, Meihui Li, Cheng Liang, Mingyu Li, Zhihui Li, Qin Xu, Lei Zhang, Wantao Chen

**Affiliations:** ^1^Department of Oral Maxillofacial Head and Neck Oncology, Ninth People’s Hospital, Shanghai Jiao Tong University School of Medicine, Shanghai, China; ^2^Shanghai Key Laboratory of Stomatology, Shanghai Research Institute of Stomatology, National Clinical Research Center of Stomatology, Shanghai, China; ^3^Beijing Advanced Innovation Center for Big Data-Based Precision Medicine, Beihang University, Beijing, China; ^4^College of Life Sciences, Qilu Normal University, Jinan, China; ^5^Shandong Children’s Microbiome Center, Qilu Children’s Hospital of Shandong University, Jinan, China; ^6^Qingdao Human Microbiome Center, The Affiliated Central Hospital of Qingdao University, Qingdao, China; ^7^Shandong Provincial Key Laboratory of Oral Tissue Regeneration, School of Stomatology, Shandong University, Jinan, China; ^8^Department of Human Microbiome, School of Stomatology, Shandong University, Jinan, China; ^9^State Key Laboratory of Microbial Technology, Shandong University, Qingdao, China; ^10^School of Information Science and Engineering, Shandong Normal University, Jinan, China

**Keywords:** 16S rRNA gene, microbiota, OSCC, cross-sectional study, salivary and OSCC bacteriome

## Abstract

Oral squamous cell carcinoma (OSCC) is affected by the interaction between oral pathogen and holobionts, or the combination of the host and its microbial communities. Studies have indicated the structure and feature of the microbiome in OSCC tissue and saliva, the relationships between microbiota and OSCC sites, stages remain unclear. In the present study, OSCC tissue (T), saliva (S) and mouthwash (W) samples were collected from the same subjects and carried out the microbiome study by 16S sequencing. The results showed the T group was significantly different from the S and W groups with the character of lower richness and diversity. Proteobacteria were most enriched in the T group at the phylum level, while Firmicutes were predominant in groups S and W. At the genus level, the predominant taxa of group T were *Acinetobacter* and *Fusobacterium*, and for group S and W, the predominant taxa were *Streptococcus* and *Prevotella*. The genera related to late stage tumors were *Acinetobacter* and *Fusobacterium*, suggesting microbiota may be implicated in OSCC developing. Both compositional and functional analyses indicated that microbes in tumor tissue were potential indicator for the initiation and development of OSCC.

## Introduction

As one of the largest habitats of microorganisms in human body, the oral cavity contains more than 1000 different kinds of microbes (Lamont et al., 2018). Within the oral cavity, the distinct habitats of hard and soft tissues contributed to the heterogeneous microbial communities which are formed depending on the oral anatomic location (Dewhirst et al., 2010). The dysbiosis of oral microenvironment was proved to be the cause of or closely related with a number of oraldiseases (Dewhirst et al., 2010; Ahn et al., 2012; Han and Wang, 2013; He et al., 2015), such as dental caries, periodontal disease, periapical and pulp diseases, and oral cancer (Takahashi and Nyvad, 2008; Zaura et al., 2009; Chen et al., 2010; Yost et al., 2015). Oral microorganisms and their metabolites also influence remote tissues and organs through the digestive tract and periodontal pocket ulceration (Pizzo et al., 2010), which were reported to associate with digestive system diseases (Warren et al., 2013), nervous system diseases (Riviere et al., 2002), cardiovascular diseases (Fåk et al., 2015), diabetes (Fardini et al., 2010), rheumatoid arthritis (Zhang et al., 2015), premature birth (Mendz et al., 2013) and were discovered in some malignant tumors (Meurman, 2010; Farrell et al., 2012). Therefore, the oral microecology is an important contributor of human health or diseases.

Oral cancer is one of the most prevalent cancers globally. More than 90% of oral cancer is squamous cell carcinoma (OSCC), which developed from the oral mucosa (Kademani, 2007). With surgery-based treatment, the 5-year survival rates of OSCC are only approximately 60.0%, which is greatly impact the patients’ quality of life (Jemal et al., 2010). OSCCs could be induced by alcohol and tobacco consumption, residual root and rough artificial tooth stimulation, poor oral hygiene etc., which has become a clinical challenge due to the high prevalence, recurrent relapse, unpredictable metastasis, oral and maxillofacial damage (Hooper et al., 2007; Crozier and Sumer, 2010). During the process of oral carcinogenesis, the local microenvironment is altered and in the meantime the microbiota composition were changed (Rivera and Venegas, 2014). The oral pathogens and the metabolites induced including nitrosamine and acetaldehyde were reported to stimulate inflammation, promote the cellular proliferation and inhibit the cellular apoptosis (Hooper et al., 2009). The composition analysis of oral microbiota between OSCC patients and healthy volunteers showed the anaerobic bacteria and acid-resistant bacteria including *Porphyromonas gingivalis*, *Streptococcus mitis* and *Fusobacterium* were increased in OSCC tissues, while Firmicutes (mainly *Streptococcus*) and Actinobacteria (mainly *Rothia*) were significantly decreased (Hooper et al., 2006, 2007). In a comparison of healthy subjects, *Capnocytophaga gingivalis*, *Prevotella melaninogenica*, and *Streptococcus mitis* were increased in the saliva of OSCC patients (Smruti et al., 2012).

Oral microbiota are potential biomarkers for the development and prognosis of OSCC. The oral pathogens, *P. gingivalis* and *F. nucleatum*, are reported to facilitate cancer progression by establishing chronic inflammation and disrupt the local immune response by secreting virulence factors such as FimA and FadA adhesins (Whitmore and Lamont, 2014). The detection of *P. gingivalis* or *F. nucleatum* are promising indicators of a poor prognosis. Besides, the divergence and richness of saliva microbiota increase significantly in oral leukoplakia and OSCC (Hu et al., 2016). The overall shift of oral microbiota is another promising diagnostic index for OSCC. Bacteria related to resistance to chemotherapy or radiotherapy are therapeutic targets in the treatment of OSCC (Sonis, 2017). However, studies on the taxonomic characteristic of OSCC tissues and saliva samples are still inadequate.

To investigate the character of microbiota in different stages of OSCC and the relationship between OSCC tissue and saliva, we carried out the oral microbiome study on the resected tumor tissue, saliva samples. In the present study, 30 subjects were analyzed and compared based on 16S rRNA gene sequencing. Phylogenetic Investigation of Communities by Reconstruction of Unobserved States (PICRUSt) was applied to infer and compare the potential role of microbiota from different samples of OSCC.

## Materials and Methods

### Ethics Statement

This study was approved by the Institutional Review Board of Ninth People’s Hospital, Shanghai Jiao Tong University School of Medicine (ethical approval number: 2016144). All methods were performed according to relevant guidelines and protocols, including any relevant details. Written informed consent was obtained for each participant.

### Sample Collection

Samples in this study were obtained from the sharing platform for the tissue sample and bioinformatics database of oral maxillofacial tumors^1^. 30 patients with different stages of cancer were enrolled without chemotherapy or radiotherapy. Oral cancer tissue samples were dissected from the site of the tumor during surgery, and the diameter of each sample was larger than 3 mm. Saliva and mouthwash liquid were collected pre surgery and before breakfast, a mouth rinse was performed twice with 20 ml of 0.9% saline to avoid contamination by cell debris, and the liquid from the second wash was collected into a 50 ml test tube. Saliva was collected into a 50 ml test tube after mouthwash (Corrêa et al., 2017). All samples were stored at -80°C within 20 min.

### DNA Extraction

DNA extraction was performed with a TIANamp Micro DNA Kit (TIANGEN BIOTECH CO., LTD.), following the protocol from a previous study (Guan et al., 2016). A total of 10 ml saliva, 10 ml mouthwash, and 5 mg OSCC tissues were used for the bacterial DNA extraction. For saliva and mouthwash samples, the pallet was transferred to a 1.5 ml Eppendorf tube after centrifugation. Then, the tube was incubated at 56°C for 60 min with Buffer GA and proteinase K. The tube was incubated for another 10 min at 70°C with Buffer GB and carrier RNA stock solution. The entire lysate was transferred into Spin Column CR2 (with a 2 ml collection tube) after adding 200 μl of ethanol, and the contaminants were removed by centrifugation with 500 μl Buffer GD and 600 μl Buffer PW. The pure DNA was eluted with 50 μl Buffer TB and collected into a new 1.5 ml Eppendorf tube. The sample was stored at -20°C before 16S rRNA gene amplification. For tissue samples, all the specimens were treated at same time, they were incubated with Buffer GA and proteinase K for 60 min until fully resolved. The following steps were the same as saliva and mouthwash DNA extraction procedures.

### PCR and 16S rRNA Gene Sequencing

The amplification of a V1-V2 hypervariable region of the 16S rRNA gene was performed with universal primers 27F: 5′-AGAGTTTGATCMTGGCTCAG-3′ and 338R: 5′-GCTGCCTCCCGTAGGAGT-3′ which also contained Illumina adapter sequences. Barcodes were attached to the 5′ terminus of the forward primers to multiplex the samples during sequencing. The PCR was performed in a total volume of 25 μL with 20 ng of DNA sample and 25 pmol of each primer with 2 × Taq PCR MasterMix (Tiangen, Beijing, China). The reactions were initially denatured at 95°C for 10 min, 6 cycles of denaturation for 45 s at 92°C, 50°C annealing for 30 sec and 72°C extension for 1 min, followed by 20 cycles of denaturation for 45 s at 92°C, annealing for 30 s at 68°C and extension 30 s at 72°C, with a final elongation for 9 min at 72°C. The concentration and purity of PCR products were examined with a NanoDrop2000 spectrophotometer (Thermo Fisher Scientific Inc., Wilmington, MA, United States). Purification of PCR products was performed with VAHTSTM DNA Clean Beads (Vazyme Biotech) according to the manufacturer’s instructions, and the purified PCR products were pooled afterward with equal nano mole. Sequencing of the 16S V1-V2 region of PCR products was performed by Illumina MiSeq platform (Illumina Incorporate, CA, United States).

### Sequencing and Statistical Analysis

FLASH (Fast Length Adjustment of SHort reads) method described by Magoč and Salzberg is a software tool to find the correct overlap between paired-end reads and extend the reads by stitching them together (Magoč and Salzberg, 2011), it was adopted for the joining and quality filtering of 16S rRNA gene paired-end sequencing data set. The Quantitative Insights Into Microbial Ecology (QIIME, version 1.9.1) software suite was used for sequence analysis, following the QIIME tutorial^2^. The split_libraries_fastq.py command was then applied demultiplexing of Fastq sequence data. *De novo* models of Usearch61 were applied for the removal of chimeric sequences. Clusters of filtered sequences were referenced to the 2013 Green genes (13_5 release) ribosomal database’s 97% reference dataset^3^ with pick_open_reference_otus.py command. UCLUST was used to cluster unmatched sequences into *de novo* OTUs at 97% similarity. Taxonomic annotation of all OTUs was achieved by the RDP classifier from the reference data set of Green Genes. OTUs with relative abundance lower than 0.02% or present in less than 20% of samples were excluded. With the alpha diversity and rank abundance function from the QIIME pipeline, rarefaction curves and rank abundance curves were calculated from OTU tables using the alpha_rarefaction.py command. UPGMA clustering (Unweighted Pair Group Method with Arithmetic mean, also known as average linkage) was used to calculate the hierarchical clustering from population profiles with the prevalence and abundance of taxa based on the distance matrix of OTU abundance. By using the QIIME package, we obtained the results in a Newick formatted tree. Reads did not match with the amplicon sequence amplification were discarded to remove the contamination by host genomic DNA.

### Statistical Analysis

The OTU table of raw counts was normalized to an OTU table of relative abundance values. Same types of taxa were agglomerated at the phylum, class, order, family and genus level. Non-parametric Wilcoxon test was used to compare the biodiversity between classified groups. The test about the alpha diversity of each groups adopt Kendall’s Tau and Spearman’s rank correlation coefficients. We used unweighted and weighted Unifrac distance of even OTU samples to perform Principal Coordinate Analyses (PCoA) and ANOSIM was used to analyze the difference among groups. LDA Effect Size (LEfSe) was performed to find out the differentially enriched taxa between groups. The functional prediction of microbiota was done with PICRUSt (Langille, Zaneveld et al., 2013). Only reads identified in closed reference picking (Greengenes 13_5 database) were used for the PICRUSt analysis, OTUs were picked at a 97% percent identity. The reference genome coverage of samples was also calculated using weighted Nearest Sequenced Taxon Index (NSTI) score with the -a option in the predict metagenomes.py script. The graphical representation of the results was performed by STAMP (Parks and Beiko, 2010).

## Results

A total of 4,606,312 raw reads were generated from OSCC tissue (T), saliva (S) and mouthwash (W) groups as shown in Table 1, data of four samples from the T group were excluded due to insufficient reads. 2,507,184 clean reads were generated with an average of 29153.302 (std.dev. 932.637) for each subject, which covered 97.6% across samples on average. The percent of chimeras was 11.3%. As a result, 12 phyla, 40 families, 63 genera and 533 OTUs were annotated among the whole samples (also see Supplementary Figures S1A,B for information about class and order level).

**Table 1:** Patient demographic data.

Variable	Total (*N* = 30)	Tissue (*n* = 26)	Saliva (*n* = 30)
**Age, years**			
Average (range)	58 (33–80)	60 (45–80)	58 (33–80)
**Tumor stage**			
I-II	25	22	25
III-IV	5	4	5
**Tumor site**			
Cheek	6	6	6
Gingiva	4	4	4
Oropharynx	7	6	7
Tongue	10	7	10
Others	3	3	3
**Tlcohol**			
Yes	7	6	7
No	23	20	23
**Tobacco**			
Yes	8	7	8
No	22	19	22

*Age, tobacco and alcohol consumption, and tumor information were provided in the form. The others option of tumor site included temple, mouth floor and maxillary.*

**FIGURE 1:**
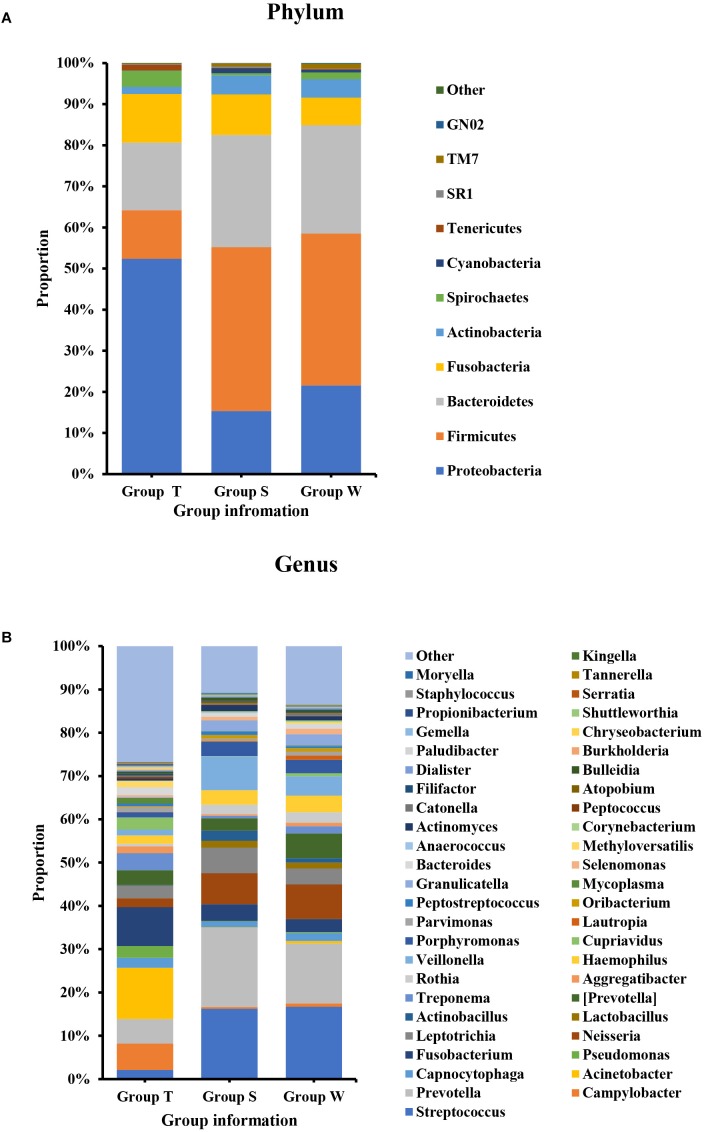
Bacterial composition on phylum **(A)** and genus **(B)** levels by OTU analysis. S, saliva group; W, oral wash group; T, tumor tissue group. The leading phyla were Firmicutes, Bacteroidetes, Proteobacteria, Fusobacteria, and Actinobacteria. On the genus level, the predominant genera were *Prevotella, Streptococcus, Veillonella, Leptotrichia, Fusobacterium*, etc.

As shown in Figure 1, the microecological composition of group S was similar to group W, but different from group T. T group enriched more Proteobacteria, which contributed to 52% of the taxonomic units, followed by Bacteroidetes (16%), Fusobacteria (12%), Firmicutes (12%) and Actinobacteria (2%) on phylum level (Figure 1A). However, Firmicutes was the most predominant phylum for groups S and W which accounting for 40% and 37% respectively, followed by Bacteroidetes (S: 27%, W: 26%), Proteobacteria (S: 15%, W: 21%), Fusobacteria (S: 10%, W: 7%) and Actinobacteria (S: 5%, W: 4%) (Figure 1A). At family level (Supplementary Figure S1C), *Enterobacteriaceae* (14%) and *Moraxellaceae* (12%) were higher abundant in group T, followed by *Fusobacteriaceae* (9%) and *Campylobacteraceae* (6%). For S and W groups, the top ranked taxa were *Prevotellaceae* (S: 18%, W: 14%), *Streptococcaceae* (S: 16%, W: 17%), *Veillonellaceae* (S: 9%, W:6%) and *Neisseriaceae* (S: 8%, W: 9%). At the genus level (Figure 1B), the most predominant taxa in group T were *Acinetobacter* (12%) and *Fusobacterium* (9%), followed by *Campylobacter* (6%) and *Prevotella* (6%). For groups S and W, genera *Streptococcus* (S: 16%, W:17%) and *Prevotella* (S:18%, W:14%) accounted for the majority of bacteria, which were only accounted for 2% and 6%, in group T.

### Alpha and Beta Diversity Analysis on OSCC Tissue, Saliva and Mouthwash Groups

**FIGURE 2:**
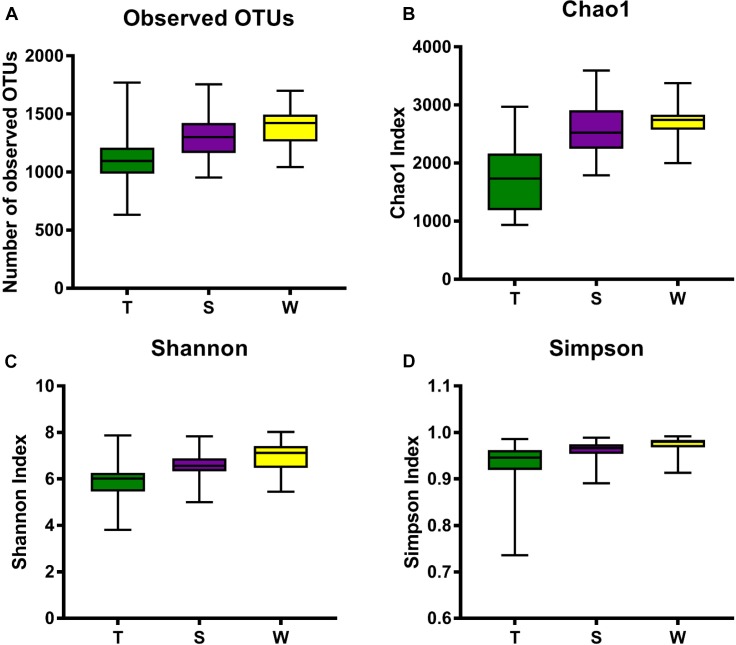
Alpha diversity analysis against all three groups. Box plots of Observed OTUs **(A)**, Simpson index **(B)**, Shannon’s index **(C)** and Chao 1 index **(D)** are shown. The indexes of groups S and W were higher than group T from the four plots, indicating that the S group and W group had higher alpha diversity than the T group.

**FIGURE 3:**
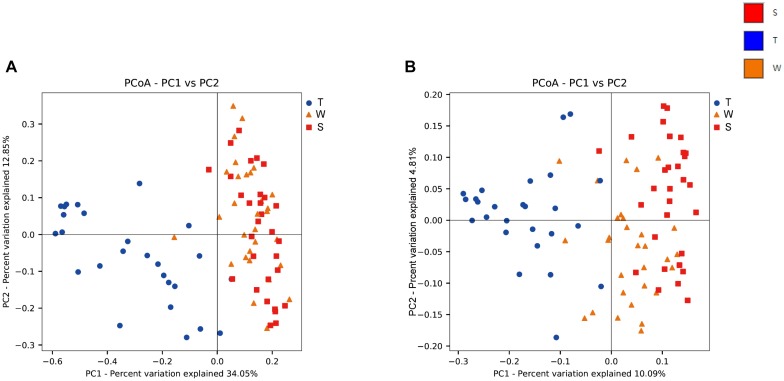
Beta diversity analysis among groups. Weighted **(A)** and unweighted **(B)** PCoA plot with respect to the bacterial abundance and composition. In the weighted PCoA, PC1 explained 34.05% of the variation, and PC2 explained 12.85% of the variation. In the unweighted PCoA, PC1 accounted for 10.09% of the variation, and PC2 accounted for 4.81% of the variation.

The alpha diversity of OSCC tissue, saliva and mouthwash groups was calculated at a maximum depth of 26,605 sequences per sample based on the Observed Species (Figure 2A), Chao1 index (Figure 2B), Shannon’s index (Figure 2C) and Simpson index (Figure 2D). Results showed the alpha diversity in OSCC tissue was significantly lower than that in saliva and mouthwash while the taxonomic richness within-samples was more similar between groups S and W (Figure 2A–D). The beta diversity analysis by principal coordinates analysis (PCoA) was shown in Figure 3. The results showed that the phylogenetic distance significantly separated group T from group W and S in both the weighted (Figure 3A) and unweighted Unifrac (Figure 3B), the difference between the group W and S was not statistically significant. ANOSIM analysis showed that R equalled to 0.75 for weighted Unicfrac (*p* = 0.0001) when we compared T group with S and W groups. The above results showed group T was significantly different from group W and group S in terms of diversity within samples and similarity between samples.

A Venn diagram was used to identify the unique and common genera among all three groups (Supplementary Figure S1D). The results showed that at the genus level, taxa of group S were fully covered by group W, and 35 genera were shared by all three groups in total. There were 13 unique genera from group T, namely, *Deinococcus*, *Rubrobacter*, *Parabacteroides*, *Chryseobacterium*, *Sphingobacterium*, *Staphylococcus*, *Lachnospira*, *Faecalibacterium*, *Megamonas*, *Phascolarctobacterium*, *Burkholderia*, *Comamonas*, and *Serratia*. There were two unique genera in group W, *Schwartzia* and TG5 from Dethiosulfovibrionaceae.

### Taxonomic Level Comparison of OSCC Tissue, Saliva and Mouthwash Groups

**FIGURE 4:**
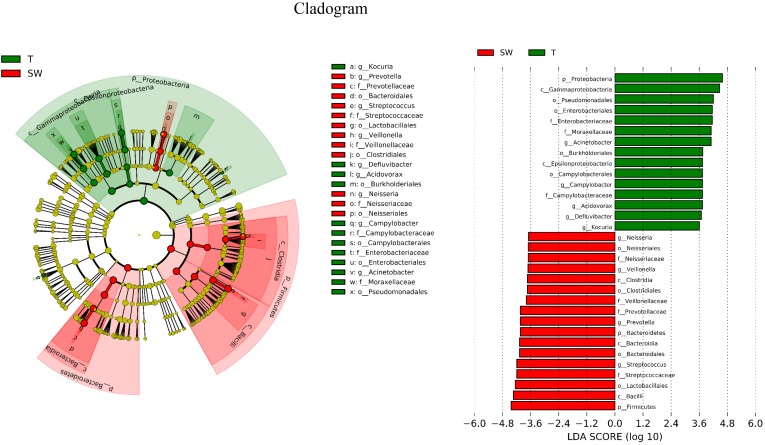
Biomarker analysis. LEfSe analysis between OSCC tissue and saliva, biomarkers from the phylum level to genus level are indicated on the right.

LDA Effect Size (LEfSe) is an algorithm to identify high-dimensional biomarkers from multiple groups. In this study, LEfSe analysis was used to identify the different composition of microbiota and to trace significant biomarkers (LDA > 2). As shown in Figure 4, the significant taxa at different levels were exhibited. The enriched taxa in OSCC tissue were aggregated under Proteobacteria, mainly in family *Campylobacteraceae*, *Enterobacteriaceae* and *Moraxellaceae*. At the genera level, the most enriched genus in OSCC was *Acinetobacter* followed by *Campylobacter*. The enriched taxa in saliva and mouthwash samples were from Firmicutes and Bacteroidetes, expect members of *Neisseriales*. *Prevotellaceae, Streptococcaceae, Veillonellaceae* were more abundant at family level. The genera *Prevotella* and *Streptococcus* were most enriched in saliva and mouthwash (Supplementary Figure S2).

### Functional Prediction of Predominant Taxa of OSCC

**FIGURE 5:**
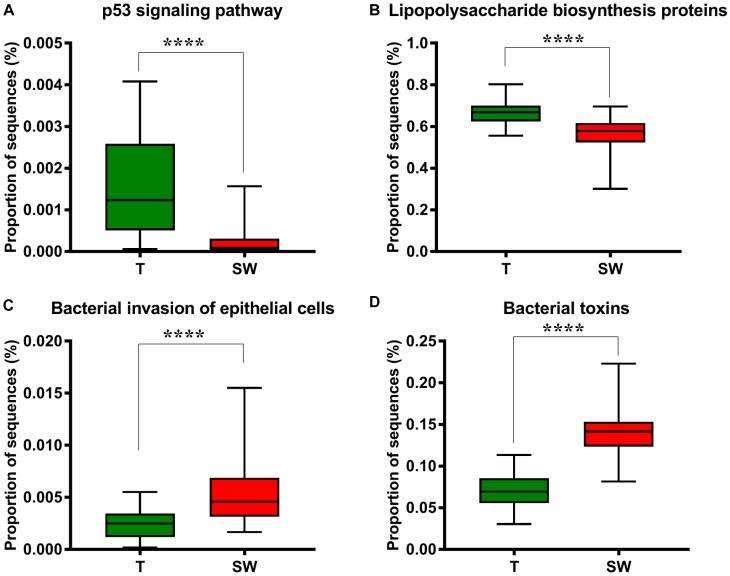
Box plot showing the significantly different KEGG items between group T and groups S and W. Group T had a higher proportion of sequences in TP53 pathways **(A)** and lipopolysaccharide biosynthesis proteins **(B)**; Groups S and W had a higher proportion of sequences in bacterial invasion of epithelial cells **(C)** and bacterial toxins **(D)**.

We used Phylogenetic Investigation of Communities by Reconstruction of Unobserved States (PICRUSt) to infer the KEGG pathways between the microbiota of group T and groups S and W. A significant difference was found in the following KEGG pathways: the OSCC microbiome had a higher abundance in the p53 signaling pathway (Figure 5A, *p* = 4.31E-05) and LPS biosynthesis proteins (Figure 5B, *p* = 3.09E-09); the S and W groups were more enriched in the bacterial invasion of epithelial cells (Figure 5C, *p* = 4.89E-08) and bacterial toxins (Figure 5D, *p* < 1E-10).

### Microbial Characteristics Analysis Among OSCC Stage and Location

**FIGURE 6:**
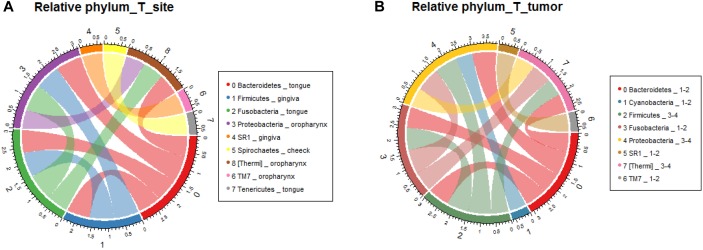
Relative analysis of taxa against OSCC tumor sites. The correlations between the microbial profile and tumor sites were performed at the phylum level **(A)** and genus level **(B)**, respectively.

**FIGURE 7:**
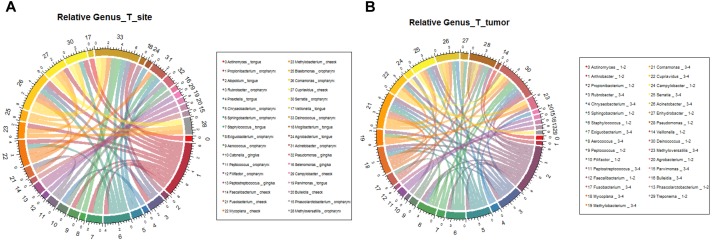
Relative analysis of taxa against OSCC tumor stages. The correlations between the microbial profile and tumor stages were showed on phylum **(A)** and genus **(B)** levels.

In order to verify the relationship between microbial composition and OSCC in different parts, we first carried out microecological composition analysis. At the phylum level, the high abundance of Bacteroidetes and Fusobacteria was detected in tongue tumors, Firmicutes was enriched in gingiva sites and Proteobacteria was enriched in oropharynges (Figure 6A, correlation >0.6, *p* < 0.05). At the genus level, the most abundant taxa of each tumor site were *Prevotella* (tongue), *Acinetobacter* (oropharynx), *Pseudomonas* (gingiva) and *Fusobacterium* (cheek) (Figure 7A, correlation >0.8, *p* < 0.05). The results indicate that the bacteria associated with tumorigenesis may be different in different parts of OSCC.

We further analyzed the relationship between microbiota composition and different stages of OSCC. In the early tumor stage, the relative abundance of Bacteroidetes and Fusobacteria were significantly higher, while in the late tumor stage, the significant enriched taxa were Firmicutes and Proteobacteria (Figure 6B, correlation >0.6, *p* < 0.05). At the genus level, the most enriched genera in the early OSCC stage were *Campylobacter* and *Prevotella*, while *Acinetobacter* and *Fusobacterium* were more enriched in the late OSCC stage (Figure 7B, correlation >0.8, *p* < 0.05). The shared taxa of different tumor sites (Supplementary Figures S3A,B) and tumor stages (Supplementary Figures S3C,D) were analysed, and no significantly enriched taxa were found. We performed the relative analysis of taxa against alcohol and smoking, but the relativeness of the taxa was all below 0.4 (Supplementary Figure S4).

## Discussion

Our study is a pilot report on the microbiota consistency and diversity in tumor tissues, saliva and oral wash samples from the same patient with OSCCs. In previous studies, some models of microbe infection and oral tumourigenesis have already been established. For instance, HPV is a cause of oral cancer through the Rb pathway (Hu et al., 2016). *Candida albicans* has been reported to have a higher prevalence in patients with OSCCs and leukoplakia. Infections by *P. gingivalis* and *F. nucleatum* have been proven to cause cancer through pathways of MMP9 and upregulation of cytokines such as TNF-α, IL-1β, and IL-6 (Herrero et al., 2003; Whitmore and Lamont, 2014; Jahanshahi and Shirani, 2015). However, the understanding of the relationship between the shift in oral microbiota and OSCC pathogenesis is still not fully established (Hu et al., 2016). Studies have been performed to analyze the microbial diversity between OSCC patients and healthy subjects using saliva or cancer tissue samples, but the relationship between microbiota in OSCC and oral cavity fluid was not clear.

The OSCC microbiota is spatially divided into two subgroups: the superficial and deep portions of the tumor tissue. The oral wash samples were included in this study as a supplement to the saliva and shed, during the sample collection, saliva was collected after rinse of the whole mouth, we supposed that mouthwash may have better contacts with tumor site, however, they showed similar properties to saliva microbiota (Figure 1, 3). Thus, the saliva and mouthwash data were combined in the following analysis. Several studies have reported an increase in *Fusobacteria* in OSCC (Schmidt et al., 2014), which was consistent with the high level of *Fusobacteria* in our research, especially in the late tumor stage. The results showed that Proteobacteria was the most predominant phyla in OSCC tissue, and a previous study indicated that the relative abundance of Proteobacteria in oral cavity mucosa was less than 20% (Schmidt et al., 2014). The percentage of Firmicutes was lower in OSCC tissue than in the S and W groups. The relative abundance of *Proteobacteria* in group T was as high as 52% (Figure 1A), the richness was largely contributed by *Acinetobacter* and *Campylobacter*, but this was not observed in other studies. Since the Proteobacteria subgroups are mainly anaerobic and facultative anaerobic bacteria (Ringel et al., 2015), the inner tissue would be a suitable microenvironment for the colonization and growth of these bacteria. At the genera level, extremely low abundance of *Streptococcus* and *Rothia* were observed (Figure 1B), which was in agreement with previous research (Pushalkar et al., 2012). The high levels of *Fusobacterium*, *Acinetobacter* and *Campylobacter* were thought to be associated with local infection and inflammation. The top 10 taxa that differentiate OSCC tissue from saliva were *p_Proteobacteria, c_Gammaproteobacteria, o_Pseudomonadales, o_Enterobacteriales, f_Moraxellaceae, g_Acinetobacter, o_Burkholderiales, c_Epsilonproteobacteria, o_Campylobacterales* and *g_Campylobacter* (Figure 4, ranked by LDA value from large to small). There is an immune suppression in a patient with advanced cancer, for instance, the accumulation of *Pseudomonadales* is related to several oral diseases, and *Enterobacteriales* and *Acinetobacter* are often observed in infections in the intestinal and urinary tract (Fouts et al., 2012; Peters et al., 2016); we speculated that the increase in these taxa in OSCC tissue was a signal of immune system depletion.

Our results indicated that there were unique genera in cancer tissue that were not detected from saliva or mouthwash (Supplementary Figure S1D). *Deinococcus* is known for its robust survival ability against ionizing radiation and oxidative stress. Species of the *Deinococcus* genus utilize their highly conserved helicase RecQ to precisely recover the genome from damage (Cox and Battista, 2005). Members of the *Rubrobacter* genus have similar antioxidant activities (Pavlopoulou et al., 2016). There were also genera found to be infectious, such as *Chryseobacterium*, *Sphingobacterium*, *Staphylococcus*, *Serratia*, and *Burkholderia*. For instance, in the *Chryseobacterium* genus, *C. meningosepticum* and *C. indologenes* are more commonly observed in human infections, and they usually cause meningitis and pneumonia, respectively, especially in patients with an impaired immune system (Nordmann and Poirel, 2002). On the other hand, we also noticed that all of these bacterial groups were typically involved in nosocomial infections, which were possibly attached during incision. Fecal bacteria such as *Parabacteroides*, *Lachnospira*, *Faecalibacterium*, *Megamonas*, and *Phascolarctobacterium* were also detected in the cancer tissue group, some of which were found to be more enriched in colon-rectal cancer (Kverka et al., 2011; Chen et al., 2012; Thomas et al., 2016; Zeng et al., 2016). The presence of these unusual taxa probably worsens the local inflammation of the OSCC inner micro environment.

To study the potential roles of microbiota in OSCC tissue and saliva sample, we performed a series of functional analyses (Wang and Ganly, 2014; Yang et al., 2018). By applying PICRUSt pathway analysis, we examined the capability of microbiota in epithelial cell invasion, bacterial toxin production, LPS synthesis protein and the p53 signaling pathway (Figure 5A–D). Overall, based on the proportion of sequences, group T had more sequences related to functions affecting the p53 signaling pathway and genes for LPS synthesis, while groups W and S were better at penetrating the epithelial cell and producing bacterial toxins. LPS may act as an effector molecule in shift oral epithelial cell to cancer (Gholizadeh et al., 2017). The p53 tumor suppressor gene is well known in oral cancer and mutated in 50% of oral cancer patients, the p53 signaling pathway is essential for regulation of cell cycle progression, differentiation, DNA repair and apoptosis (Sinevici and O’sullivan, 2016). In Greathouse’s study (Greathouse et al., 2018), they established the microbiome-*TP53* gene interaction in human lung cancer tissue, and the higher abundance of certain taxa, including *Acidovorax*, were associated with *TP53* mutation in squamous cancer cells. Perera et al. (2018) suggested that compositional studies showed inconsistency among results, and functional predications were useful tools to examine the bacteriome in OSCC. In our study, the functional predication indicated that in OSCC tissues, the microbiota were more involved in LPS synthesis and escape of host cell cycle arrest, which were potential risk factors for OSCC, while in saliva, the microbiota functions were more enriched in penetrating cells and secreting toxins, which worsened the micro-environment. Considering that the functional analysis in 16S rRNA gene sequencing is based on bacteria at the genera level by targeting variable regions, which could not reflect the bacterial gene function and activity very precisely, metagenomic sequencing and co-culture with cell lines are needed in future studies (Wang et al., 2015).

## Conclusion

In conclusion, this cross-sectional study illustrated the comparison between microbiota in OSCC and saliva samples collected from the same subjects. In OSCC tissue, the most abundant taxa were *Acinetobacter* and *Fusobacterium*, they were also found predominantly in the late stage of OSCC, their ability of causing infection and local inflammation were potential facilitator of OSCC progress. The microbiota composition in mouth wash samples were similar to saliva samples, but both of them were distinct from OSCC tissue. The PICRUSt pathway analysis suggested the role of OSCC and saliva microbiota, respectively. There were several limitations of this study: (1) Restricted by the resolution of the 16S technique, the similarity of OTUs was set to 97%, which was not accurate enough to differentiate members at the species level with limited functional information, and the amplification biases may lead to inaccuracy of the result; (2) In our study, we only included 30 subjects, which was still a small sample size. An enlarged group size will be needed in future validation studies. Strategies such as whole-genome shotgun sequencing and metabolomics will be used to achieve a more detailed analysis. Longitudinal research will be performed to study the relationship between oral microbiota shift and OSCC progress.

## Data Availability

The datasets used and/or analyzed during the current study available from the corresponding author on reasonable request. Sequence files and metadata for all samples used in this study have been deposited in SRA (PRJNA528843).

## Author Contributions

WC and LZ designed the study. WC, QX, MiL, and ZhL collected all the saliva and tissue samples. ZZ, JY, BC, QF, and MeL performed the measurements and data analysis. ZZ, LZ, and WC wrote the manuscript. All authors have read and critically revised the manuscript.

## Conflict of Interest Statement

The authors declare that the research was conducted in the absence of any commercial or financial relationships that could be construed as a potential conflict of interest.

## Footnotes

1 http://mdl.shsmu.edu.cn/OMNDB/page/home/home_en.jsp

2 http://qiime.org

3 http://greengenes.secondgenome.com/

## Supplementary Material

FIGURE S1 Compositional analysis of taxa at the class level **(A)**, order level **(B)** and family level **(C)**. **(D)** Venn diagram showing the number of shared and unique species of each group at the genus level.

FIGURE S2 The most enriched genera in group T or S and W group.

FIGURE S3 The shared taxa of different tumor sites **(A,B)** and tumor stages **(C,D)**.

FIGURE S4 The relative analysis of taxa against smoking and drinking history of patients, the relation with OSCC tissue **(A)** and saliva **(B)** sample were showed. ^∗^Means between 0.05 and 0.1, ^∗∗^stands for less than 0.05.
