# Supplementary figures and images for: Compositional and Functional Analysis of the Microbiome in Tissue and Saliva of Oral Squamous Cell Carcinoma
Source: Front Microbiol. 2019 Jun 26;10:1439. doi: 10.3389/fmicb.2019.01439 (PMC6607966; doi:10.3389/fmicb.2019.01439)

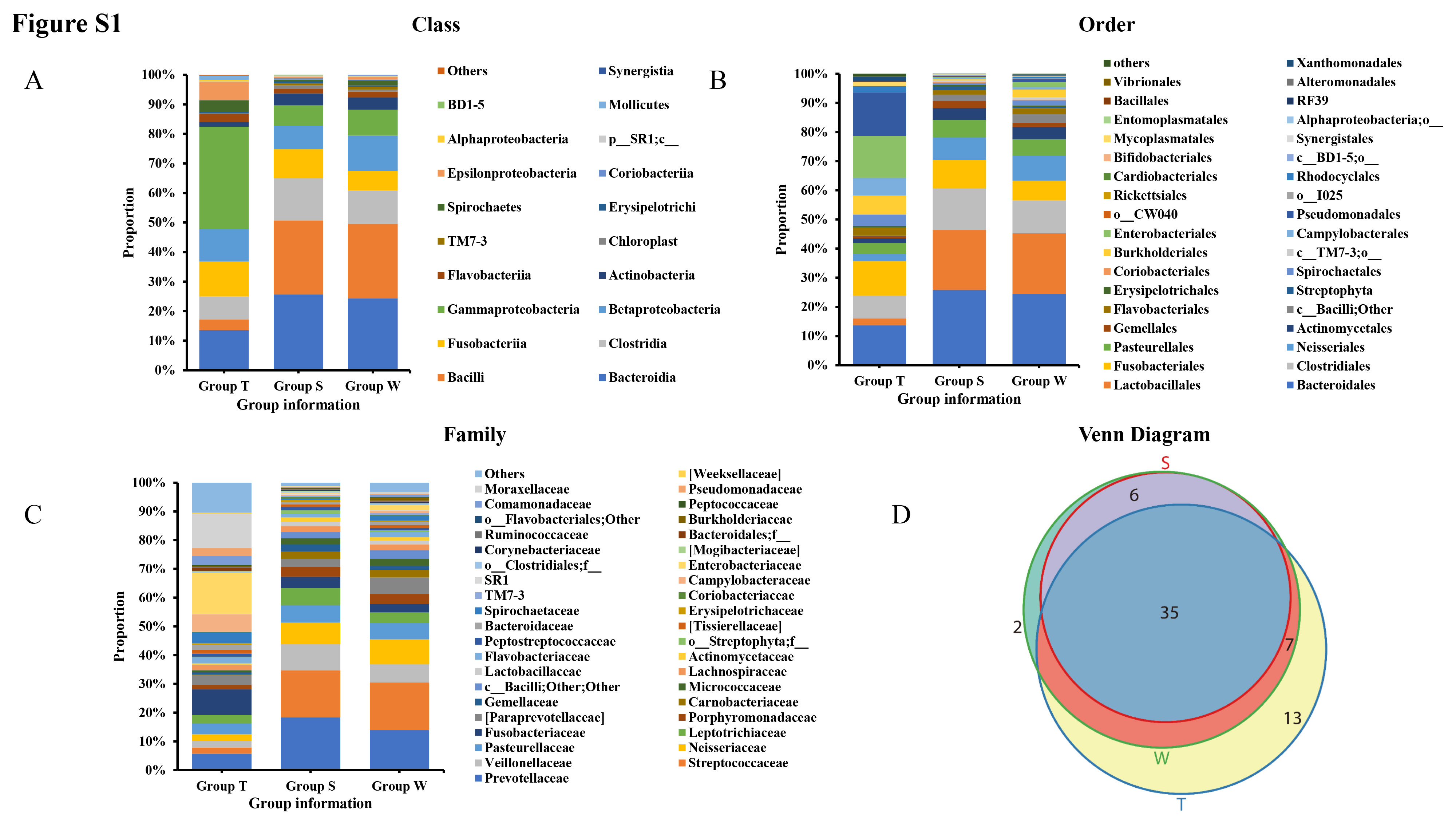

Supplement: FIGURE S1 — Compositional analysis of taxa at the class level (A), order level (B) and family level (C). (D) Venn diagram showing the number of shared and unique species of each group at the genus level. [file Image_1.JPEG]

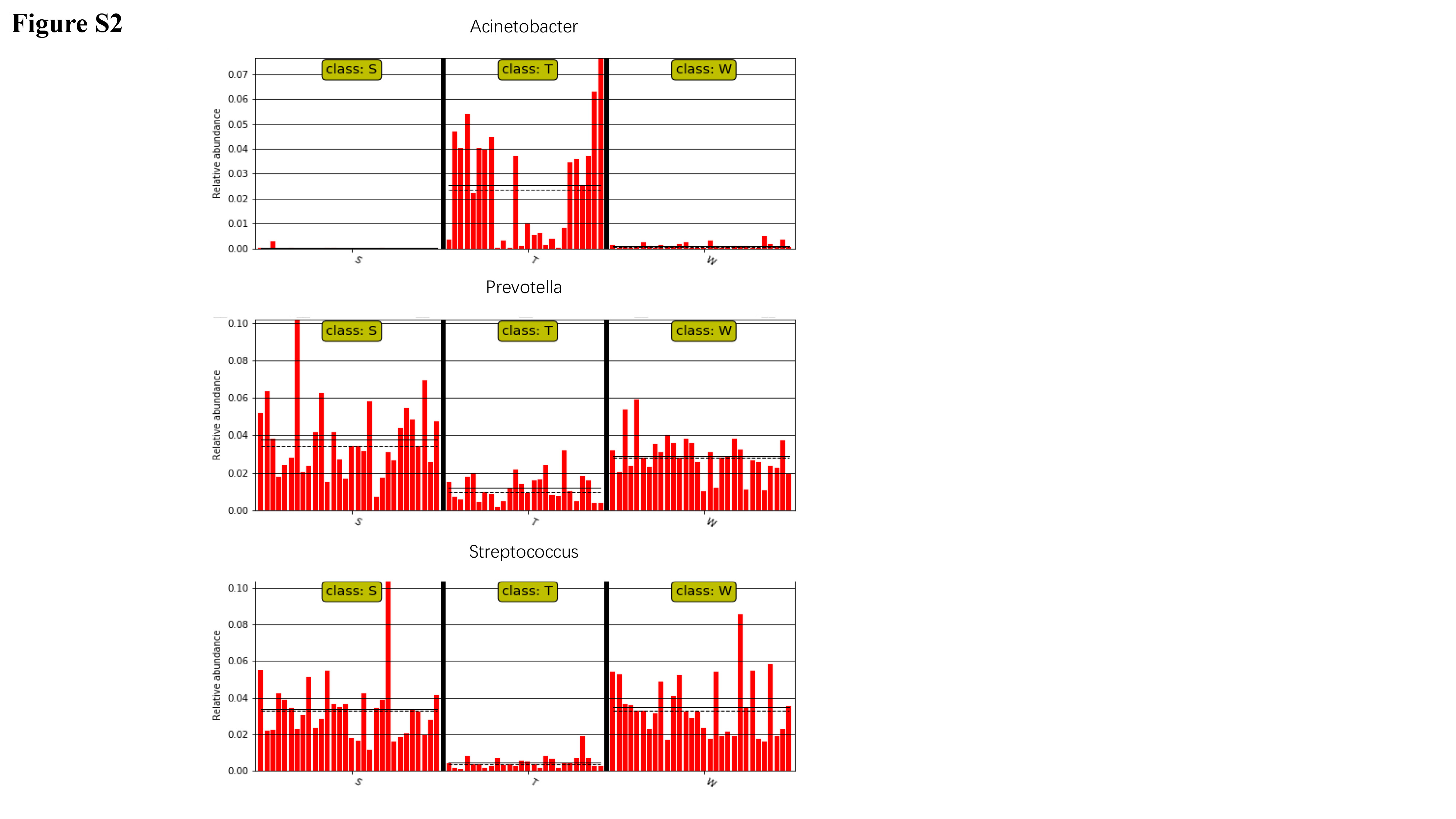

Supplement: FIGURE S2 — The most enriched genera in group T or S and W group. [file Image_2.JPEG]

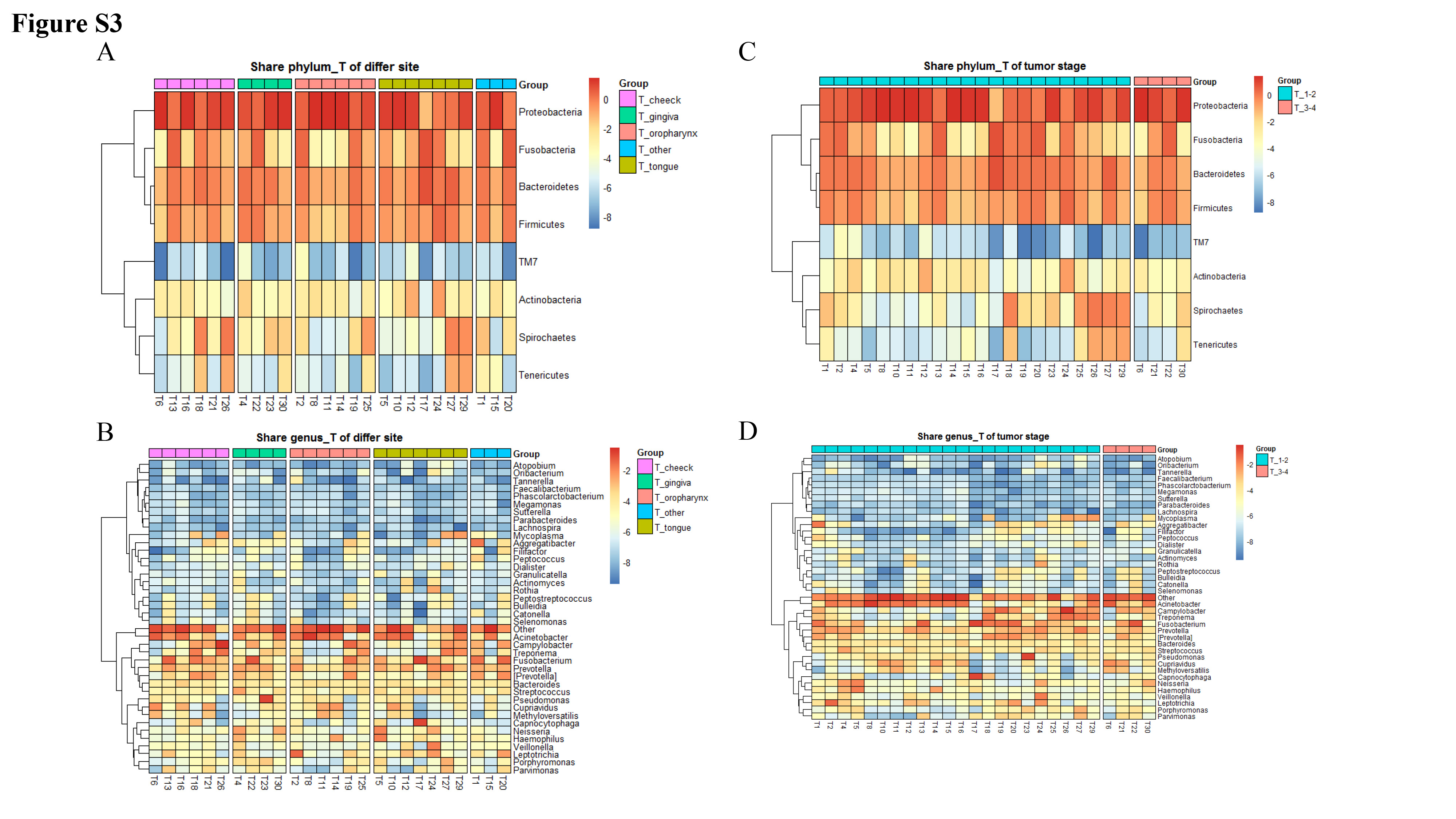

Supplement: FIGURE S3 — The shared taxa of different tumor sites (A,B) and tumor stages (C,D). [file Image_3.JPEG]

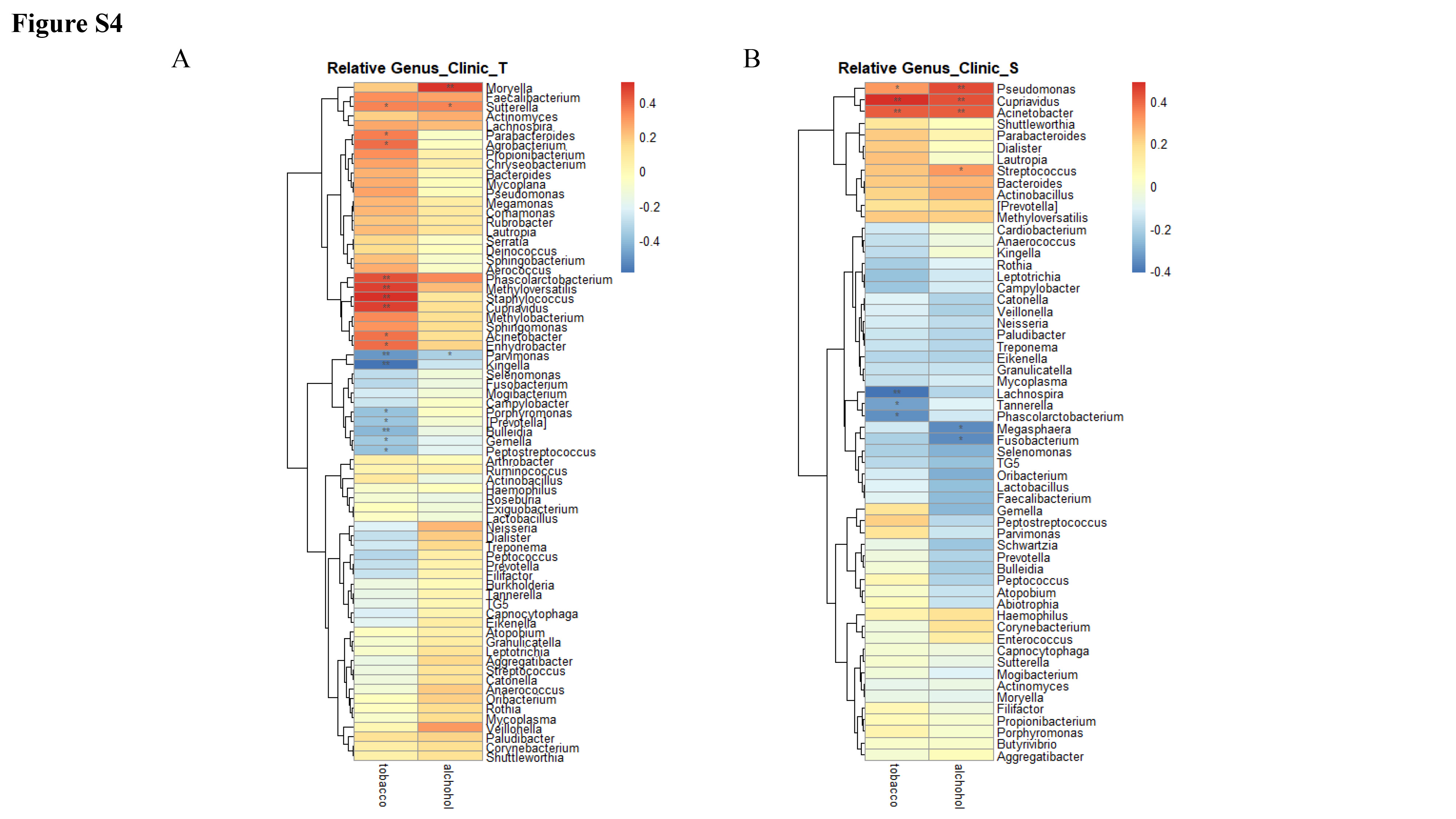

Supplement: FIGURE S4 — The relative analysis of taxa against smoking and drinking history of patients, the relation with OSCC tissue (A) and saliva (B) sample were showed. ∗Means between 0.05 and 0.1, ∗∗stands for less than 0.05. [file Image_4.JPEG]
